# A Functional and Structural Mongolian Scots Pine (*Pinus sylvestris* var. *mongolica*) Model Integrating Architecture, Biomass and Effects of Precipitation

**DOI:** 10.1371/journal.pone.0043531

**Published:** 2012-08-22

**Authors:** Feng Wang, Véronique Letort, Qi Lu, Xuefeng Bai, Yan Guo, Philippe de Reffye, Baoguo Li

**Affiliations:** 1 Key Laboratory of Plant-Soil Interactions, Ministry of Education, Department of Soil and Water Science, China Agricultural University, Beijing, China; 2 Ecole Centrale of Paris, Department of Applied Mathematics, Chatenay-Malabry, France; 3 Institute of Desertification, Chinese Academy of Forestry, Beijing, China; 4 Liaoning Sand Stabilisation and Afforestation Institute, Fuxin, China; 5 CIRAD, UMR AMAP, Montpellier, France; DOE Pacific Northwest National Laboratory, United States of America

## Abstract

Mongolian Scots pine (*Pinus sylvestris* var. *mongolica*) is one of the principal tree species in the network of Three-North Shelterbelt for windbreak and sand stabilisation in China. The functions of shelterbelts are highly correlated with the architecture and eco-physiological processes of individual tree. Thus, model-assisted analysis of canopy architecture and function dynamic in Mongolian Scots pine is of value for better understanding its role and behaviour within shelterbelt ecosystems in these arid and semiarid regions. We present here a single-tree functional and structural model, derived from the GreenLab model, which is adapted for young Mongolian Scots pines by incorporation of plant biomass production, allocation, allometric rules and soil water dynamics. The model is calibrated and validated based on experimental measurements taken on Mongolian Scots pines in 2007 and 2006 under local meteorological conditions. Measurements include plant biomass, topology and geometry, as well as soil attributes and standard meteorological data. After calibration, the model allows reconstruction of three-dimensional (3D) canopy architecture and biomass dynamics for trees from one- to six-year-old at the same site using meteorological data for the six years from 2001 to 2006. Sensitivity analysis indicates that rainfall variation has more influence on biomass increment than on architecture, and the internode and needle compartments and the aboveground biomass respond linearly to increases in precipitation. Sensitivity analysis also shows that the balance between internode and needle growth varies only slightly within the range of precipitations considered here. The model is expected to be used to investigate the growth of Mongolian Scots pines in other regions with different soils and climates.

## Introduction

Land degradation and desertification in arid, semiarid and dry sub-humid areas is a global issue with increasing importance, and also an immense problem causing environmental degradation and the ever increasing loss of natural resources in China [1]. To combat desertification, the Chinese government started the implementation of the Three-North Shelterbelt Programme, which is composed of a network of shelterbelts and tree plantations across the entire region in North China since 1978 [2]. Mongolian Scots pine (*Pinus sylvestris* var. *mongolica*) is one of the principal tree species in the network of Three-North Shelterbelt for windbreak and sand stabilisation in China. The natural distribution of Mongolian Scots pines is mainly in the Hulun Buir sandy lands, northeast China. Because it has high tolerance to cold, drought, soil infertility and grows naturally in the sandy land, this species had been introduced to the edge of sandy lands in northern China to protect nearby lands from moving sand dunes since the 1950s. In these arid and semi-arid regions, the competition for the limited water resources is a major factor influencing the sensitive balance of these ecosystems. In that context, model-assisted analysis of water balance in Mongolian Scots pine, in relation to canopy development and ecophysiological dynamics, is of value for better understanding of its role and behaviour within these shelterbelt ecosystems [3], [4].

Functional-structural plant models (FSPMs) are effective tools for studying the growth and development of plants by integrating three-dimensional (3D) plant architecture with eco-physiological processes [5], [6]. Some early FSPMs focused more on the dynamic changes of plant function in static tree architecture than on architectural development, e.g., ECOPHYS [7], [8], EMILION [9] and SIMWAL [10]. Later, LIGNUM [11], [12] and L-PEACH [13], [14] simulated tree metabolism and carbon allocation within a dynamic tree architecture. The GreenLab model is a discrete dynamic model that represents biomass production and allocation at organ scale with feedback effects of internal trophic competition on plant morphology [15]. It is thus able to simulate the plant phenotypic plasticity that results from interactions between its growth, morphological differentiation and the trophic condition of the organism [16]. The GreenLab model has been applied to crops, e.g., maize [17], wheat [18], tomato [19] and trees such as beech [20], eucalyptus [21] and Chinese pine [22].

At present, the interactions between plant growth and the environment are of primary interest when discussing the feedback processes between plant structure and function [23]. In a previous study, we modelled the 3D structure of Mongolian Scots pine using the formalism of a dual-scale automaton [24]. However, such a structural model represents only the dynamics of tree architectural development in a particular environment [25], and does not account for plant physiology and plasticity in response to environmental change (e.g. precipitation). In this study, our objective is to adapt the GreenLab model to analyse the effects of water availability on tree height, secondary growth (basal diameter) and biomass accumulation in Mongolian Scots pines. For young plantations in arid and semiarid areas, light interception was not considered as a discriminant process for pine tree growth since solar radiation was abundant and variability in terms of branch orientation was not very high. In contrast, cumulative precipitation was much less than the cumulative reference evapotranspiration in our study region [26]. Therefore, the effects of water availability could be focused upon in our study.

In this paper, we show how to couple soil water dynamic to the GreenLab model as it is applied to Mongolian Scots pine. The resulting model considers 3D architecture, plant biomass and soil water-content availability. Our goals in this paper are: a) to introduce water-use efficiency into the GreenLab model; b) to apply this adapted GreenLab model to Mongolian Scots pine; to calibrate the model; and to quantitatively validate the model using detailed measurement data; c) finally, to analyse the effects of decreases or increases in precipitation on canopy architecture and biomass accumulation of Mongolian Scots pine trees by numerical simulation.

## Materials and Methods

### Study Site and Experiments

The study area is the plantation station of the Liaoning Sand Stabilisation and Afforestation Institute, located in Zhanggutai (122°22’E, 42°43’N), Liaoning province, China. This region is adjacent to the Kerqin sandy land, which is one of the four biggest sandy lands in China. Long-term (1953–2006) mean annual precipitation is 505.9 mm, concentrated mainly between May and October. Mean annual temperature is 6.0°C with monthly mean temperatures ranging from −12.1°C in January to 24.1°C in July. The soil type is classified as aeolian sandy soil according to the Chinese classification, or arenosols according to the Food and Agriculture Organisation (FAO) classification, with 93.98%±6.00% sand, 5.49%±5.46% silt, and 0.52%±0.55% clay. The soil texture is uniform through the whole profile (0–300 cm). Pines were planted in 1 m (between-row)×1 m (within-row) spacing in the study plot. The understory is bare sandy land.

Measurements of plant structure, organ biomass and soil attributes were carried out in November, 2006, and, in August, 2007 in the same region, sampled on different trees of similar age groups. The branching patterns were derived from field observations on trees aged from four to six years: the number of branches of each branching order, their insertion angle in the vertical direction and their azimuth were recorded for 100 trees of each age-group (four, five and six year-olds). These observations were also used to assess the duration of the growth cycle and of organ functioning. The standard tree samples were selected from the plots and taken for destructive measurements for one-, two-, four-, five- and six-year-old trees in 2006 and one-, two-, three-, five- and six-year-old trees in 2007 with four replications in each tree age group. Length, diameter, fresh and dry biomasses of each “internodes” and needles inserted were recorded. Soil was sampled at depth intervals of 20 cm through the soil profile (0–300 cm). Soil mechanical composition was measured using a laser particle-size analyser (MasterSizer 2000, Malvern, UK).

### Model Description

**Figure 1:**
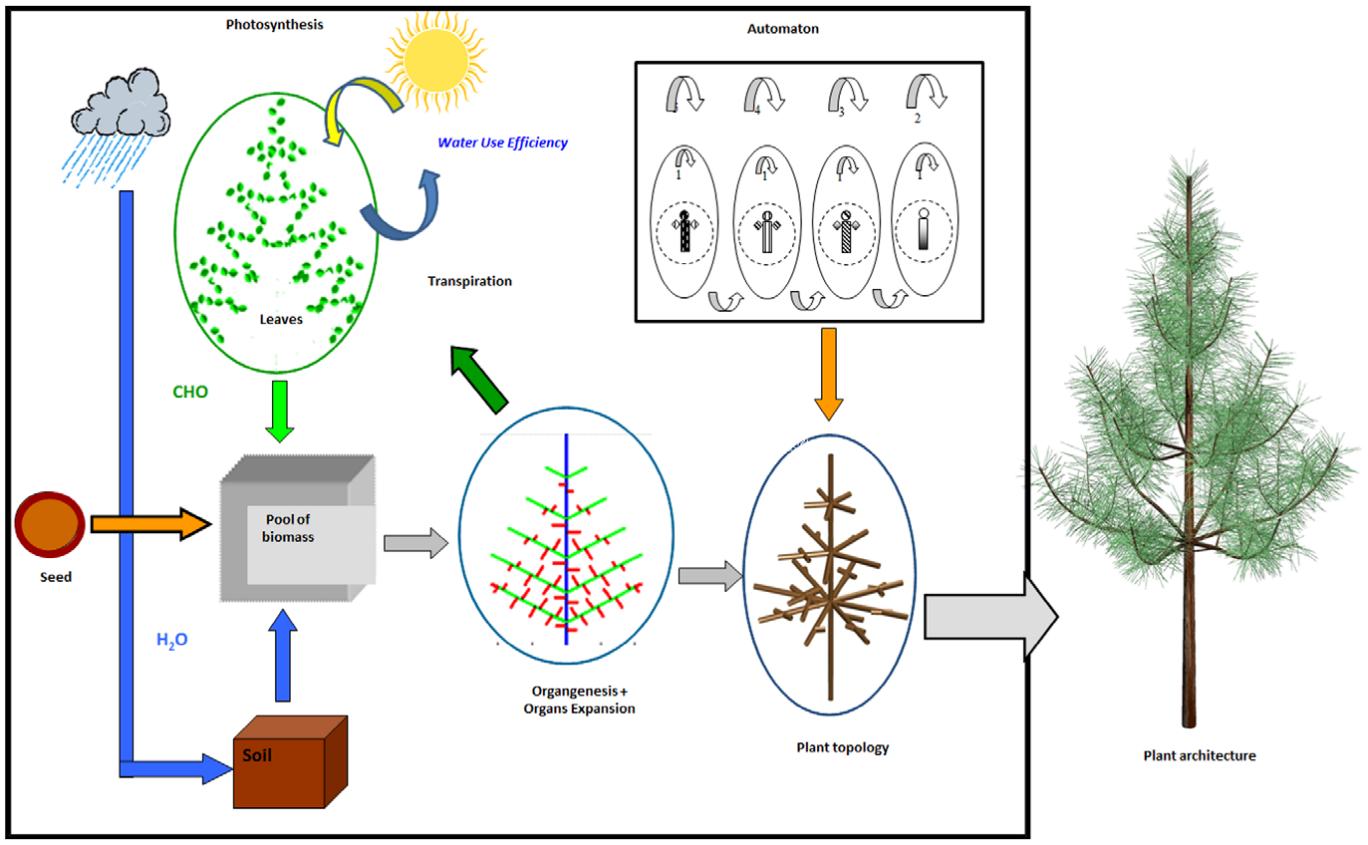
Conceptual scheme of the functional-structural tree model coupled with soil water balance for Mongolian Scots pines: the seed gives the initial pool of biomass, which is used to build organs (internodes, leaves) and thus the plant architecture. The seedlings take up water from soil for leaf transpiration and biomass production during each growth cycle. The biomass produced is a product of the amount of water transpired by plant and water-use efficiency (WUE). The biomass is stored in the common pool of reserves and is then distributed among organs, which ends the growth cycle. The plant topology, which deals with the physical connections between plant components, is constructed based on automaton rules at the organ scale. Plant architecture can be constructed by topological and geometric information, which includes the shape, size, orientation and spatial location of the components.

A new module for computing daily soil water dynamic proposed was introduced into the GreenLab model and coupled with plant canopy architecture, biomass production and allocation modules. Figure 1 shows the conceptual scheme of this model. The seed gives the initial pool of biomass, which is used to build organs (internodes, leaves) and thus the plant architecture. The seedling takes up water from soil for leaf transpiration and biomass production at each growth cycle (thermal time elapsed between the formations of successive phytomers) equal to one year. The biomass produced is derived from a product of the amount of water transpired by plant and water-use efficiency (WUE). The biomass produced is stored in the common pool of reserves and is then distributed among organs, which ends the growth cycle. The plant topology, which deals with the physical connections between plant components, is constructed based on automaton rules at the organ scale. Plant architecture can be constructed by topological and geometric information, which includes the shape, size, orientation and spatial location of the components.

**Figure 2:**
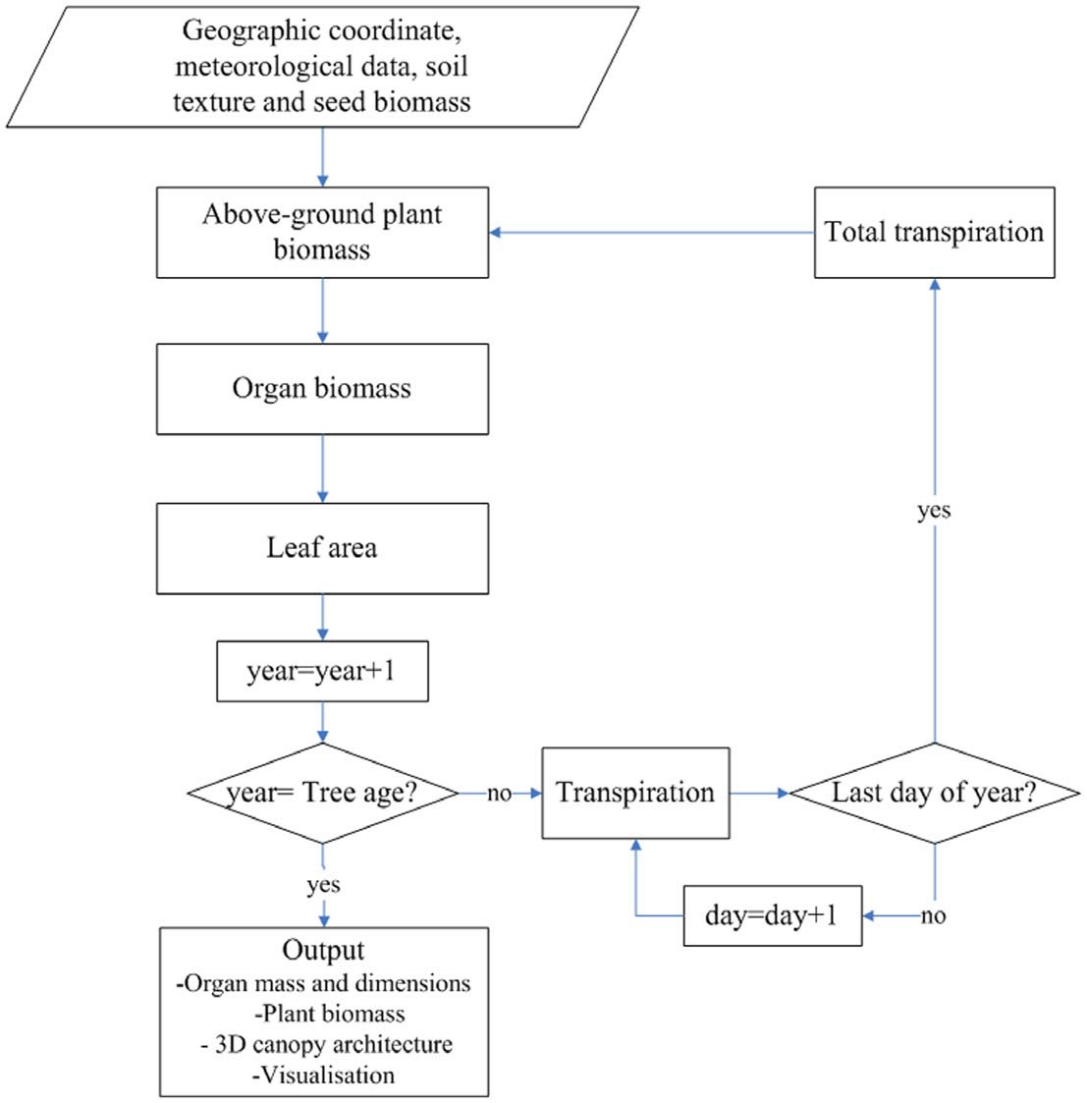
Flowchart for the functional-structural tree model coupled with soil water balance for Mongolian Scots pines. The fluxes of the model are computed on two time scales: daily for the plant transpiration and yearly for the processes of biomass production and 3D canopy development. There are interactions and feedbacks between the plant architecture (shoots) and transpiration and water absorption through leaf area index (LAI).

The processes of 3D canopy development, biomass production and allocation were simulated at a yearly time scale, as described in detail in section 2.2.1, 2.2.2 and 2.2.3. The soil water dynamic process was calculated daily, as described in detail in section 2.2.4. The model flowchart is shown in Figure 2. The model requires as input the geographic coordinate, meteorological data, soil texture and seed biomass. Finally, the model outputted the biomass of whole plant and individual organ and visualization of 3D canopy architecture. Note that only the aboveground growth is modelled in this work. This means that the model excludes the influences of root extension on water uptake.

### Plant Architecture

The developmental sequence of Mongolian Scots pine was analysed qualitatively by identification of organ types and their functioning times. Each growth unit (GU) is composed of internodes, needles, one apical bud and several lateral buds. The growth units are classified according to a botanical index called physiological age (PA), which characterises the state of meristem differentiation at growth unit initiation [27]. Each organ belonging to a given PA-based class, shares the same set of parameters that defines its functioning and its positioning in the tree’s architecture. For the tree species studied here, PA is equivalent to branching order and ranges from one for the trunk to four for the third-order branches, the maximal branching order being three in the trees sampled. Plant topology is constructed based on automaton rules at the organ scale. Plant geometry, which mainly includes the inclination and azimuth of the branch etc., is measured directly in the experiments. A detailed description of the 3D canopy architecture model can be found in [24].

### Biomass Production

By considering the plant water-use efficiency, the aboveground biomass is derived from the simulated amount of water transpired. The water-use efficiency expresses the aboveground dry matter [g] produced per unit land area [m^2^] per unit of water transpired [mm]. Many experiments have shown that the relationship between biomass produced and water consumed by a given species is highly linear [28]. The aboveground biomass production *Q*(*i*) at tree age *i* [year] is obtained from actual transpiration *T*_a_(*i*) and water-use efficiency *WUE*:
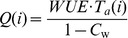
(1) where *C*_w_ is the plant average water content [%].

### Biomass Allocation

Biomass is allocated to the plant organs according to their relative sink strengths, which indicates the abilities of different kinds of organs to compete for biomass [29]. The overall demand *D*(*i*) at tree age *i* is the sum of sink strengths of all the appearing and growing organs in the aboveground plant, as shown in Eq. (2):
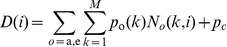
(2) where *o* represents organ type, *a* stands for needles (all needles on one growth unit are treated as a single entity), *e* stands for internode and *p_c_* stands for the sink strength of secondary growth; *M* is maximum PA of the tree, being four for the third-order branches in this study; *p*_o_(*k*) represents the relative sink strength of organ of type *o* and PA *k*; *N_o_*(*k*, *i*) represents the number of organ of type *o* and PA *k* appearing at tree age *i*.

A new organ *o* (leaf or internode) with PA *k* gains biomass in proportion to its sink strength and the ratio between the biomass supply from the previous cycle and current demand at tree age *i*, as shown in Eq. (3):
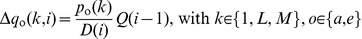
(3)


At each growth cycle, biomass allocation to organs and organ dimensions (e.g. internode length and diameter, and needle surface areas) were computed with empirical equations describing source-sink relationships and allometric rules. Relevant equations used for biomass repartition in tree stem and associated allometric rules are given in the Appendix S1. A detailed description of biomass allocation can be found in [30].

### Soil Water Dynamic

Plant root zone can be presented by means of a container in which the water content may fluctuate. Root zone depletion is used to express the water content. A soil water dynamic equation is introduced to illustrate the interactions and feedbacks between the plant architecture, function and water resources. Soil water dynamic process at a plot depends on precipitation, canopy interception, run-off, evaporation, transpiration and ground water recharge. Because these Mongolian Scots pines were young and the experimental plots were located at the edge of sandy land (Groundwater level is low), canopy interception, runoff and ground water recharge are neglected in the model. The daily water dynamic in the soil profile, expressed in terms of depletion at the end of the day *n* is:
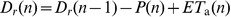
(4)

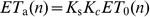
(5) where *D*_r_(*n*) is the root zone depletion at the end of day *n* [mm], *P*(*n*) represents the precipitation on the day *n* [mm], *K*_s_ is the soil water-stress coefficient, *K_c_* is the crop coefficient, equal to 1 [31]; *ET*_a_(*n*) represents actual plant evapotranspiration on the day *n* [mm] and *ET*_0_(*n*, *i*) is the reference plant evapotranspiration on the day *n* of year *i* [mm d^−1^]. The soil water dynamic process is calculated at the end of each day. The soil profile is considered as no water deficit at the beginning of growth cycle in each spring. That sets the initial value of *D*_r_(0) = 0.

The coefficient of soil water-stress *K*_s_(*n*) is calculated as proposed by [31], shown in Eq. (6):
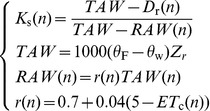
(6) where *TAW* is the amount of available water that a plant can extract from its root zone, and its magnitude depends on the soil texture and rooting depth *Z*_r_ [m]. Typical values for soil water-content at field capacity *θ*_F_ and at wilting point *θ*_w_ are given in [31] for various soil texture classes; *D*_r_(*n*) is the root zone depletion at the end of day *n* [mm]; *RAW* is the readily available soil water in the root zone, i.e. the water that a crop can extract from the root zone without suffering water stress [mm]; *r*(*n*) is the average fraction of *TAW* that can be depleted from the root zone before water-stress occurs [0–1].

### Transpiration

Actual plant transpiration *T*_a_(*i*) is the sum of daily transpiration amounts during the growth year *i* of a tree. It is calculated as proposed by [32]:
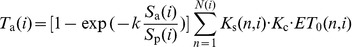
(7) where *S*_p_(*i*) is the available ground area of a tree at age *i* [m^2^]; *k* is the extinction coefficient of Beer Lambert’s law, equal to 0.5 [29]; *S*_a_(*i*) is the total functioning leaf area of a tree at age *i*, *i.e*. the sum of all individual leaf areas [m^2^]; *N*(*i*) is the maximal growth days at tree age *i*; *K*_s_(*n*, *i*) is the soil water-stress coefficient at day *n* of year *i*; crop coefficient *K_c_* is equal to 1.0 for coniferous trees [31].

Daily reference evapotranspiration *ET*_0_(*n*) is calculated by the Penman-Montieth equation [33]:
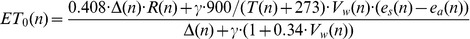
(8) where Δ(*n*) represents the slope of the vapour pressure curve, *R*(*n*) is the net radiation at the plant surface [MJ m^−2^ d^−1^], γ is the psychrometric constant [kPa °C^−1^], *T*(*n*) represents the mean daily air temperature at a height of 2 m [°C], *V_w_*(*n*) represents the mean daily wind speed at a height of 2 m [m s^−1^] and *e*_s_(*n*)-*e*_a_(*n*) represents the saturation vapour pressure deficit [kPa].

### Parameterisation and Data Sources

Daily meteorological data were obtained from a local weather station about 30 km South of the study area and included atmospheric pressure [Pa], mean air temperature [°C], maximum air temperature [°C], minimum air temperature [°C], mean relative humidity, sunshine duration [h], wind speed [m s^−1^] and precipitation [mm]. The soil texture and maximum effective rooting depth was measured in the experiment. Field water-capacity and soil water content at wilting point were obtained from [31]. Some parameters of the plant growth model, namely leaf functioning time and plant density were observed and recorded in the plantation plot. Topological and geometrical parameters were obtained from a large quantity of field data of branch number, branch angle, branch azimuth and internode length and diameter for one- to six-year-old pines. Water content of the plant *C*_w_ and specific leaf weight *ε* were calculated from organ fresh and dry biomasses and needle lengths and diameters. Organ scale coefficient *b* and shape coefficient *β* were calculated directly by analysing organ fresh biomass and dimensions.

Thirteen hidden parameters of the plant growth module, including water-use efficiency, sink strengths of needles, sink strengths of of internodes and ring repartition coefficients were estimated using a generalised least-square (GLS) method, which generates a set of parameters that minimises the sum of squares of errors between the measured data and the model outputs. The mathematical equations used in the fitting process can be found in [17]. The goodness of fit was expressed in the root-mean-squared error (RMSE) between the measured data and the corresponding model outputs. Digiplant is a software that includes an implementation of the GreenLab model and its parameter estimation [34]. It was used to estimate the parameters according to the measured data stored in so-called target files. The target files were given as input, and included the length, diameter of internodes and fresh biomass of internodes and needles of every growth unit for the six-year-old Mongolian Scots pines, and the accumulated fresh biomass of internodes and needles for one- to five-year-old pines. A common set of hidden parameters were estimated for all the target trees in parallel. Based on the generalized least squares methods for non-linear models, an adaptation of the 2-stage Aitken estimator was used, where the observations were classified into groups with respect to the type of organs (that have potentially very different size orders) and the error terms of each group, assumed mutually independent, have common unknown variance [35], [36]. The model was calibrated using the measurement data of August 2007 and validated using the measurement data of November 2006. Two datasets about pine architecture and biomass measured at different time are independent of each other. The whole model was written in C++ and implemented in Code::Blocks 8.02 platform (http://www.codeblocks.org/).

Symbols and description of the main parameters and variables used in the model are listed in Table 1.

**Table 1:** Symbols and description of the main parameters and variables used in the model for Mongolian Scots pine trees growth. Values are given only for parameters. (GU: Growth Unit, PA: Physiological Age).

*Parameters/Variables*	Description(unit)	Value
***Soil***		
*Z_r_*	Maximum effective rooting depth [m]	1
*θ_F_^†^*	Soil water content at field capacity [m^3^ m^−3^]	0.12
*θ_W_^†^*	Soil water content at wilting point [m^3^ m^−3^]	0.07
*K_c_^†^*	Crop coefficient	1
*K_s_*	Soil water-stress coefficient	
*D_r_*	Root zone depletion at the end of one day[mm]	
*ET_a_*	Actual plant evapotranspiration on one day [mm]	
*ET_0_*	Reference plant evapotranspiration on one day [mm]	
***Plant***		
*M*	Maximum PA of the tree	4
*d*	Plant density [m^−2^]	1
*B*	Scale coefficient of single GU (PA = 1, 2, 3, 4)	76.4, 163.3, 197.7, 358.0
*β*	Shape coefficient of single GU (PA = 1, 2, 3, 4)	−0.24, −0.30, −0.20, 0.14
*C_w_*	Average water content of plant	60%
*ε*	Specific leaf weight [g cm^−2^]	0.035
*k^†^*	Extinction coefficient	0.5
*WUE* ‡	Water-use efficiency [g kg^−1^]	4.5
*p_e_* ‡	Relative sink strength of “internodes” (PA = 1, 2, 3, 4)	1.37, 0.12,0.04, 0.01
*p_a_* ‡	Relative sink strength of needles (PA = 1, 2, 3, 4)	1(ref), 0.42, 0.17, 0.05
*p_c_* ‡	Relative sink strength of growth rings	11.09
*λ* ‡	Coefficient for needle influence on ring partitioning	0.03
*R_p_* ‡	Secondary sink for growth ring partitioning (PA = 2, 3, 4) [cm^−1^]	0.07, 0.01, 0.001
*T_a_*	Actual plant transpiration on one day [mm]	
*Q(i)*	Aboveground biomass produced at tree age *i* [g]	
*D(i)*	Overall biomass demand at tree age *i*	
*N_o_*	Number of organs of type *o*	
*S_p_*	Available ground area of a tree	
*S_a_*	Total functioning leaf area of a tree	
***Meteorology***		
Δ	Slope of the vapour pressure curve	
*R*	Net radiation at the plant surface [MJ m^−2^ d^−1^]	
*γ*	Psychrometric constant [k Pa °C−1]	
*T*	Mean daily air temperature at a height of 2 m [°C]	
*V_w_*	Mean daily wind speed at a height of 2 m	
*e_s_*	Saturation vapour pressure [kPa]	
*e_a_*	Actual vapour pressure [kPa]	
*P*	Precipitation [mm]	

Note: ^†^The parameter values are obtained from the reference [27].

‡ The parameter values are obtained by fitting process. The other parameters and variables are measured directly in the experiments or obtained by simple calculation. The entries with blank values are not constant in the model.

## Results

### Soil Water Dynamic Process

**Figure 3:**
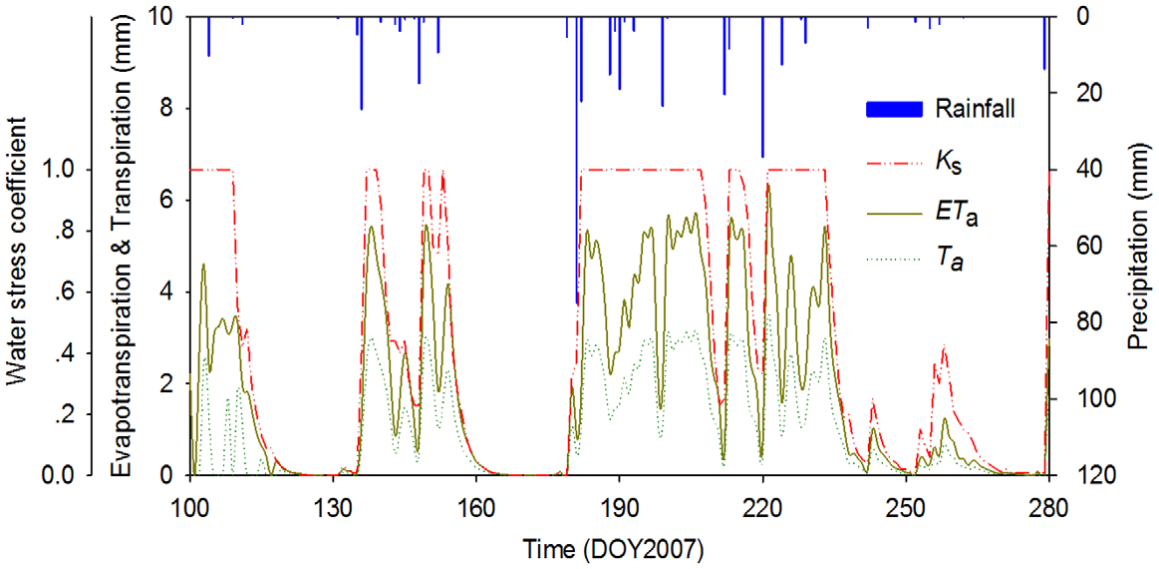
Simulation of soil water dynamic in a plot of six-year-old Mongolian Scots pines in 2007. The growth season for Mongolian Scots pine in 2007 is from 100^th^ to the 280^th^ day. Rainfall, *K*_s_, *ET*_a_ and *T*_a_ represent, respectively, daily precipitation, the simulated values of water-stress coefficient, actual evapotranspiration and actual transpiration. The soil texture is 93.98%±6.00% sand, 5.49%±5.46% silt, and 0.52%±0.55% clay and is uniform through the whole profile (0–300 cm). Maximum effective rooting depth *Z*_r_ is 1 m, soil water content at field capacity *θ*_F_ is 0.12 m^3^ m^−3^ and soil water content at wilting point *θ*_W_ is 0.07 m^3^ m^−3^. Trees were planted in 1 m (between-row)×1 m (within-row) spacing in the plot.

The simulations were performed daily for soil water dynamic process in the plantation plot. Figure 3 shows the daily precipitation, actual evapotranspiration and transpiration of six-year-old trees during the growth period in 2007. The growing season for perennial plants is considered to be between the first 5 consecutive days in spring and the last 5 consecutive days in fall with a mean daily temperature at or above 0°C, generally from mid April through mid October. The simulated results were calculated from the 100^th^ to the 280^th^ day of 2007 which is the growth season for Mongolian Scots pine. Most precipitation in the research area occurred in July and August. *K*_s_, *ET*_a_ and *T*_a_ increased significantly after each rainfall event. In the plot of six-year-old trees, annual precipitation was 412.6 mm in 2007, and actual evapotranspiration and actual transpiration were 383.5 mm and 170.9 mm, respectively.

### Model Calibration

The sink strength of needles, and primary and secondary growth of “internodes” are listed in Table 1. The results show that the sink strength of organs on branches (PA = 2, 3, 4) are significantly lower than those on the trunk for primary growth, as well as for secondary growth. It means that the trunk growth demand is dominant in Mongolian Scots pine. The ring repartition coefficient and proportion coefficient λ (as shown in Eq. (A.1)) is 0.03, which means that the relative position of needles has almost no influence on the secondary growth of internodes.

**Figure 4:**
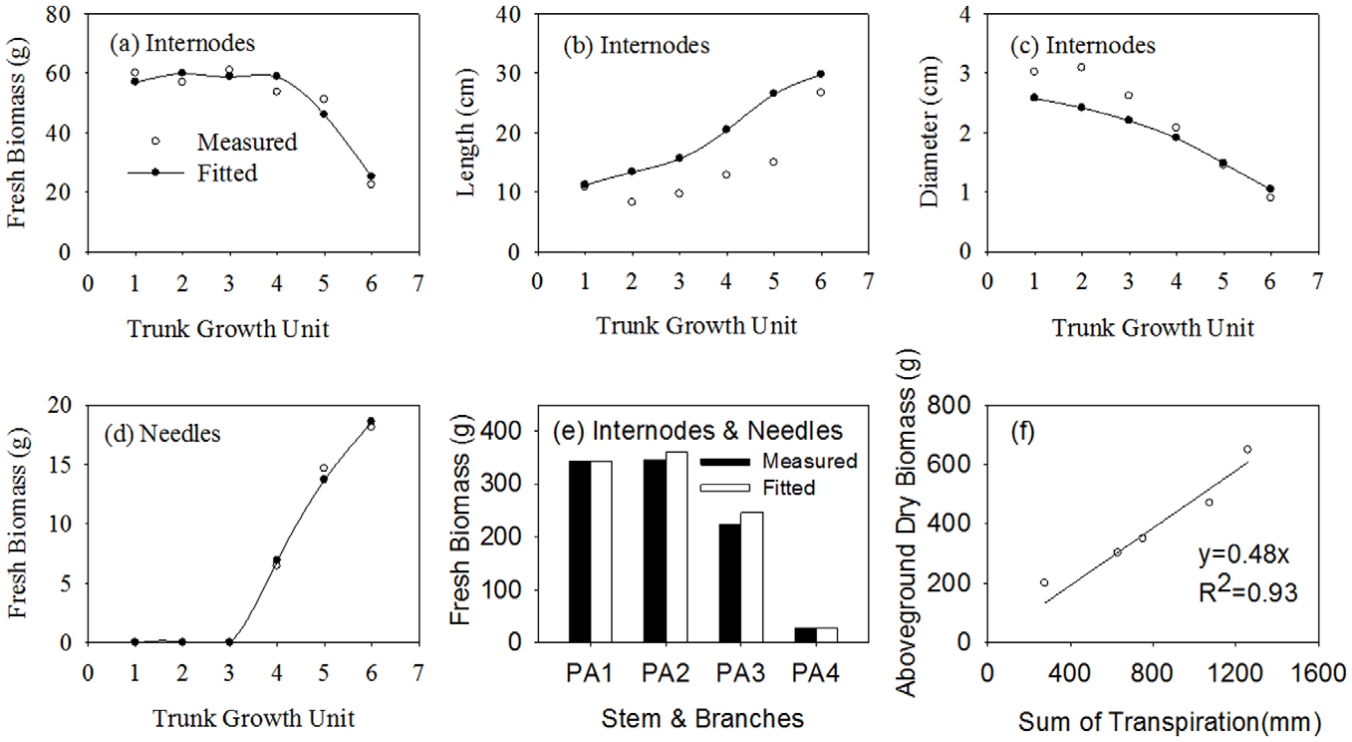
Comparisons between measured and fitted results at organ scale for six-year-old Mongolian Scots pines measured in 2007. (a) Internode fresh biomass, with RMSE = 3.72 g; (b) Internode length, with RMSE = 6.65 cm; (c) Internode diameter, with RMSE = 0.38 cm; (d) Needle biomass, with RMSE = 0.47 g; (e) Total fresh biomass of different PA (including internodes and needles), with RMSE = 10.80 g. PA1, PA2, PA3 and PA4 represent, respectively, trunk, first-order branch, second-order branch and third-order branch; (f) Linear regression between aboveground dry biomass and sum of transpiration estimated (*y* = 0.48*x*, *R* ^2^ = 0.93, *p* = 0.0001).

**Figure 5:**
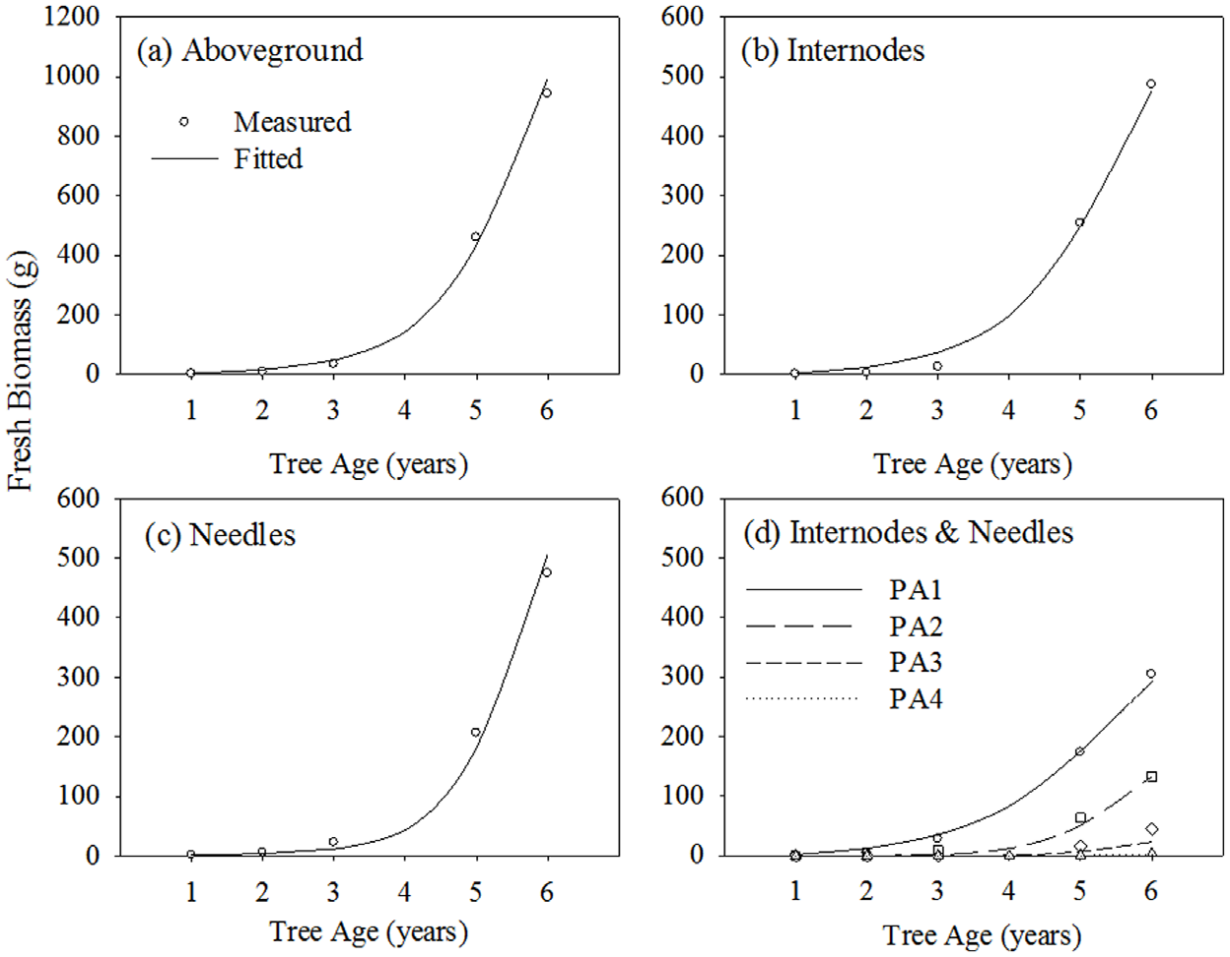
Comparisons between measured and fitted fresh biomass at plant scale for Mongolian Scots pines from one- to six-year old measured in 2007. (a) Aboveground fresh biomass, with RMSE of 26.8 g; (b) Internodes fresh biomass, with RMSE of 13.0 g; (c) Needles fresh biomass, with RMSE of 18.6 g; (d) Total fresh biomass of different PA(including internodes and needles), with RMSE = 9.4 g. PA1, PA2, PA3 and PA4, represent, respectively, trunk, first-order branch, second-order branch and third-order branch.

To evaluate the model, we compared the measurement data and the fitted results at organ scale (Figure 4) and at plant scale (Figure 5). Figure 4 (a and d) show the fitted data were consistent with measured for fresh biomass of trunk internodes and needles, with the relative errors are 7.7% and 5.6%, the root mean square root (RMSE) of 3.72 g and 0.47 g. The fitted results for internode length are higher than the observations with a relative error of 46.3%, a RMSE of 6.65 cm(Figure 4 (b)), while the relative error is 13.1% for internode diameter, a RMSE of 0.38 cm (Figure 4 (c)). Obviously, the fitted results for biomass are better than those for geometry. This may be due to continuing growth of internodes after measurements were made in August. Figure 4 (e) shows the comparison between the observations and fitted results for total fresh biomass (internodes and needles) of trunk and branches of different orders with a RMSE of 10.80 g, for which relative errors were 0.3%, 4.3%, 9.7% and −1.8%. The *WUE* value for Mongolian Scots pines is 4.5 kg m^−3^, which is obtained by fitted method in this study. Figure 4 (f) shows a significant linear relation between above-ground biomass and cumulative transpiration, with a slope for the linear regression line of 0.48, as indicated by its high *R* ^2^ (0.93). Figure 5 shows the comparison between the observations and fitted results for fresh biomass at plant scale for different ages. The model tended to overestimate above-ground biomass growth with larger relative errors for trees at age one year and two years, and decreasing to around 5% for trees at age five and six years. The overestimate declining towards the end of the simulation is consistent with the self-correcting tendency of generalised least-square method. These results indicate that the fitted data at the plant scale are better than those at the organ scale.

### Model Validation

**Figure 6:**
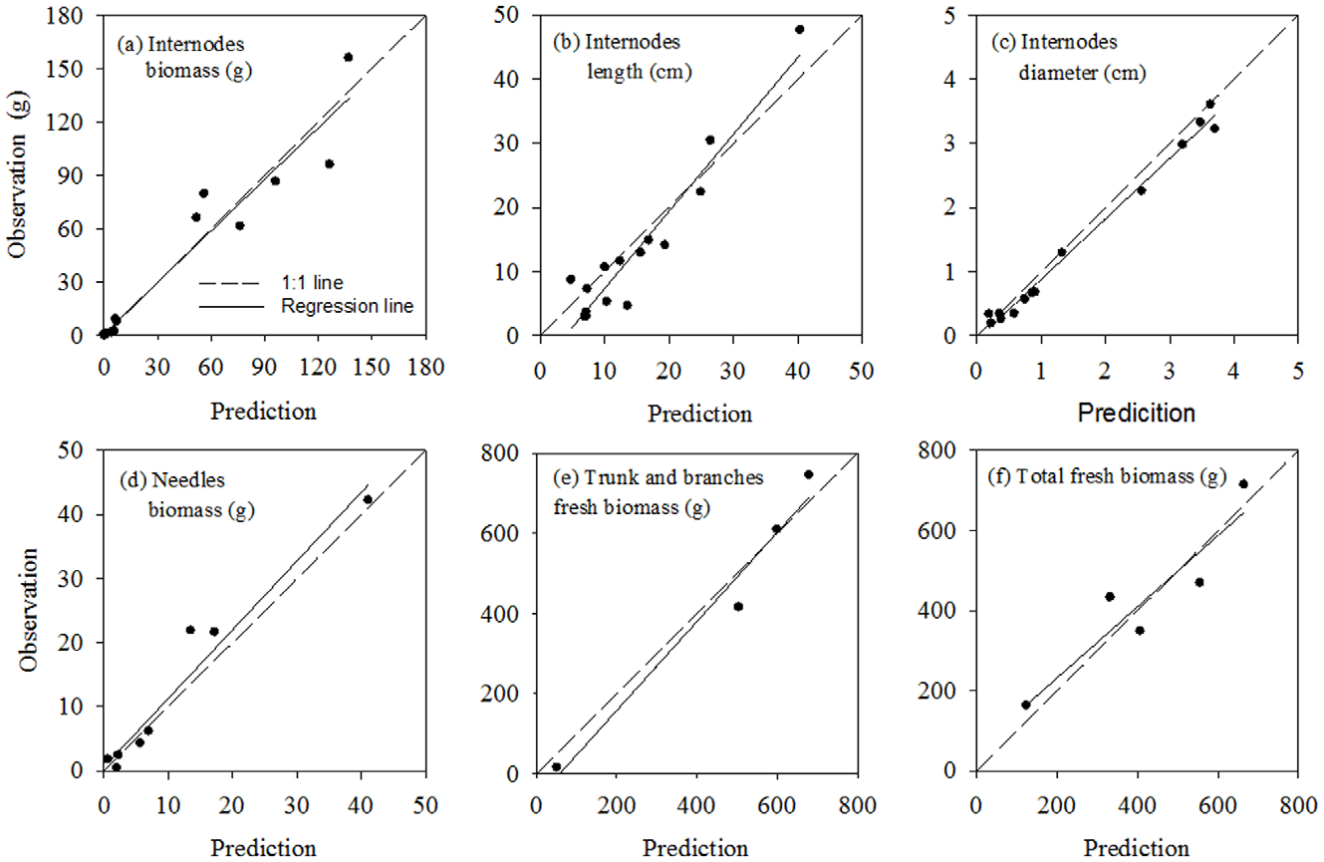
Comparison of fresh biomass, length, diameter between prediction and observation of three six-year-old pines measured in 2006. The regression equations between observations and predictions and statistical tests for each indicator are listed as following: (a) Internodes biomass, *y* = 1.01+0.96*x*, (*R* ^2^ = 0.93, *p*<0.0001, *n* = 15); (b) Internodes length, *y* = −4.64+1.20*x*, (*R* ^2^ = 0.91, *p*<0.0001, *n* = 15); (c) Internodes diameter, *y* = −0.06+0.94*x*, (*R* ^2^ = 0.99, *p*<0.0001, *n* = 15); (d) Needles biomass, *y* = 0.57+1.07*x*, (*R* ^2^ = 0.99, *p*<0.0001, *n* = 8); (e) Trunk and branches fresh biomass, *y* = −64.84+1.11*x*, (*R* ^2^ = 0.97, *p* = 0.0168, *n* = 4); (f) Total fresh biomass, *y* = 54.46+0.89*x*, (*R* ^2^ = 0.86, *p* = 0.0235, *n* = 6).

The growth of six-year-old trees was simulated by using the meteorological data from 2001 to 2006 in the calibrated model. The height and fresh biomass of a simulated tree were compared with observations for testing the accuracy of the model predictions. Table 2 shows the meteorological conditions from 2001 to 2007. The mean air temperature and precipitation were lower in 2001 than in the other years. For six-year-old trees measured at the same site conditions, the average height and above-ground fresh biomass were 105.4 cm and 1065 g in 2006, and 82.9 cm and 808.3 g in 2007. The comparisons between simulations and observations of biomass and dimension are shown in Figure 6. The relative errors of the fresh biomasses of trunk, first-order, second-order and third-order branches and of above-ground plant parts were respectively −1.6%, −8.8%, 21.6%, 238.4% and 6.7%. These relative errors increased with branching order. Obviously, the sink strength of a third-order branch was overestimated. This discrepancy may be caused by the small number of third-order branches. The estimated parameters by using GLS method are the global optimal solution. Therefore, the simulated results are better for the large number of first-order and second-order branches, but worse for the small number of third-order branches. However, compared with the trunk and first-order branches, the total fresh biomass of the third-order branches was much lower. For instance, for a six-year-old tree measured in 2006, the total fresh biomass of the third-order branches was only 0.9% of the above-ground parts. Therefore, in our experimental database, these had little influence on the simulated result of total fresh biomass of the above-ground parts.

**Table 2:** Meteorological conditions from 2001 to 2007.

	Mean air temperature(°C)	Mean soil temperature(0 cm, °C)	Total precipitation(mm)	Thermal time(≥10°C)
**2001**	7.5	9.5	309.0	3691.9
**2002**	8.4	11.3	391.2	3634.0
**2003**	8.5	11.8	508.2	3683.1
**2004**	8.7	11.5	621.4	3784.9
**2005**	7.9	10.3	586.9	3660.4
**2006**	8.2	11.4	358.9	3615.9
**2007**	9.3	12.6	412.6	3754.4

**Figure 7:**
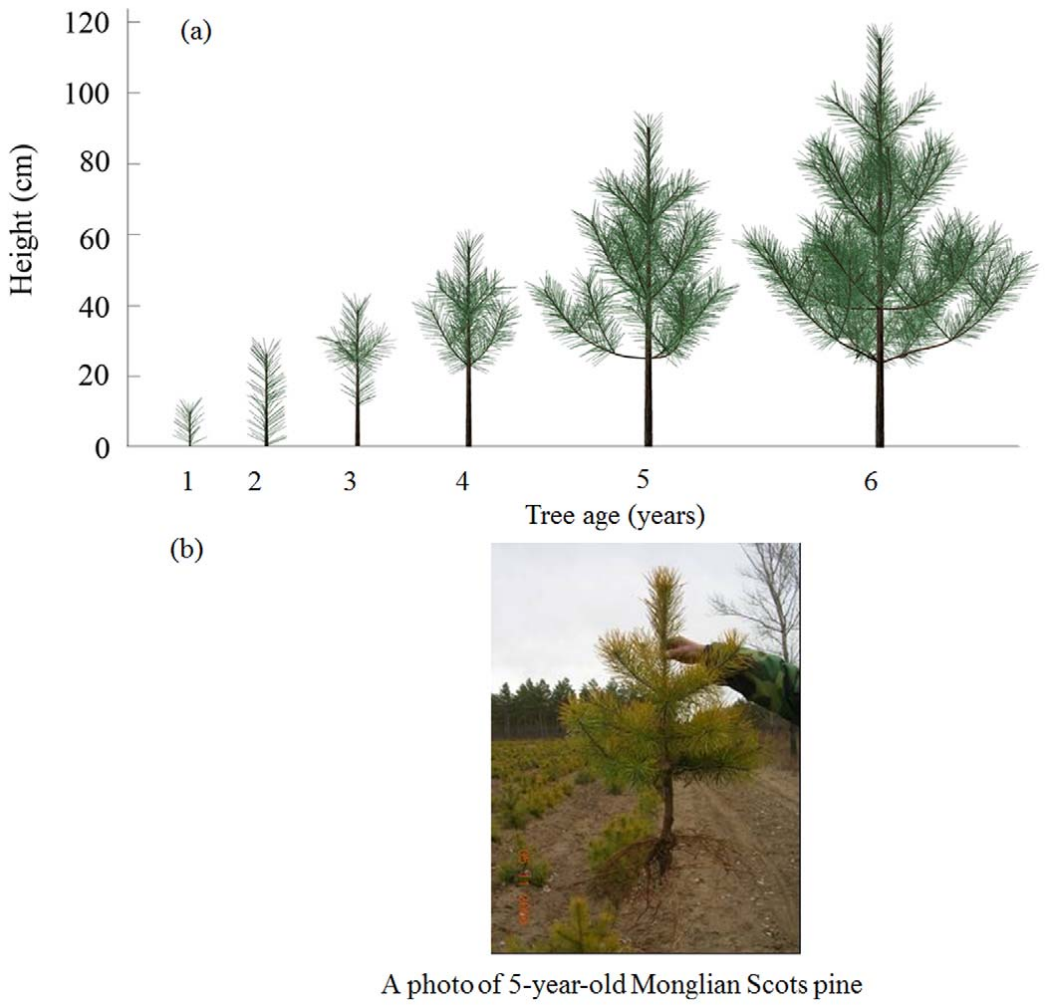
Comparison between simulated images and taken photo for Mongolian Scots pine. (a) Visualization of the 3D architecture of a Mongolian Scots pine simulated from one- to six-year old, according to local soil data and meteorological data recorded from 2001 to 2006; (b) A photo of 5-year-old Mongolian Scots pine taken in Nov, 2006.

Using the plant topological and geometrical information measured in the field, the 3D architecture of Mongolian Scots pines was visualised at successive ages from one to six years, according to local soil data and meteorological data recorded from 2001 to 2006 (Figure 7(a)). The azimuth of branches on the same node follows a uniform distribution, and the inclination of branches are between about 70° and 90° and the inclination will increase with branch age [24]. Figure 7(b) showed a photo of 5-year-old Mongolian Scots pine taken in Nov, 2006. From the Figure 7, we can see the architecture of 5-year-old Mongolian Scots pine simulated is similar with real one. The simulated tree heights were 4.4 cm, 11.3 cm, 21.0 cm, 36.4 cm, 63.3 cm and 104.6 cm from one to six years, respectively. The mean values and standard deviation of corresponding tree height measured in 2006 were 8.9±1.7 cm, 14.7±5.0 cm, 30.9±4.9 cm, 69.7±6.1 cm and 117.4±10.6 cm for one-, two-, four-, five- and six-year-old trees. Considering the individual difference of tree growth, the simulated tree heights stayed in a reasonable range.

### Sensitivity Analysis

Sensitivity analysis provides the opportunity to systematically test the model behavior and to get insights into how the simulated system operates. It has been used to determine the percentage of variation in tree height, basal diameter and biomass when precipitation changes from 50% less than actual to 50% more than actual in 5% steps.

**Figure 8:**
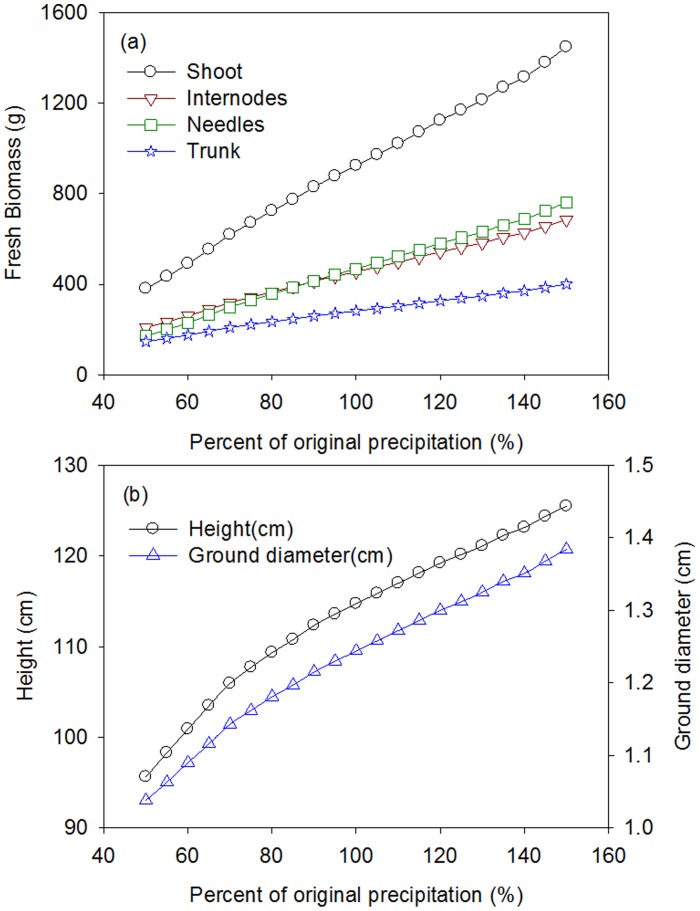
Sensitivity analysis of biomass, height and diameter for a six-year-old Mongolian Scots pine when precipitation changes from 50% less than actual to 50% more than actual in 5% steps.

The biomass results show that the internodes compartment, needles compartment and aboveground biomass responded linearly to increases in precipitation (Figure 8). A 50% decrease in daily precipitation resulted in a 59% decrease in total above-ground biomass, a 48% decrease in trunk biomass, a 17% decrease in tree height and a 17% decrease in basal diameter. A 50% increase in daily precipitation resulted in a 57% increase in total above-ground biomass, a 42% increase in trunk biomass, a 9% increase in tree height and an 11% increase in basal diameter. The effect of precipitation changes is more pronounced on tree biomass than on tree height or on basal diameter.

The proportion of biomass partitioned between internodes and needles varied from 1.22 to 0.90, which is small compared with the variation in the range of precipitation. However, there was a change in the dominant compartment: the internode biomass was higher than needle biomass for very low precipitations, while the result was the reverse for precipitations greater than 90% of the reference value. The variation trends for internode and needle biomasses were generally consistent with those for total biomass.

**Figure 9:**
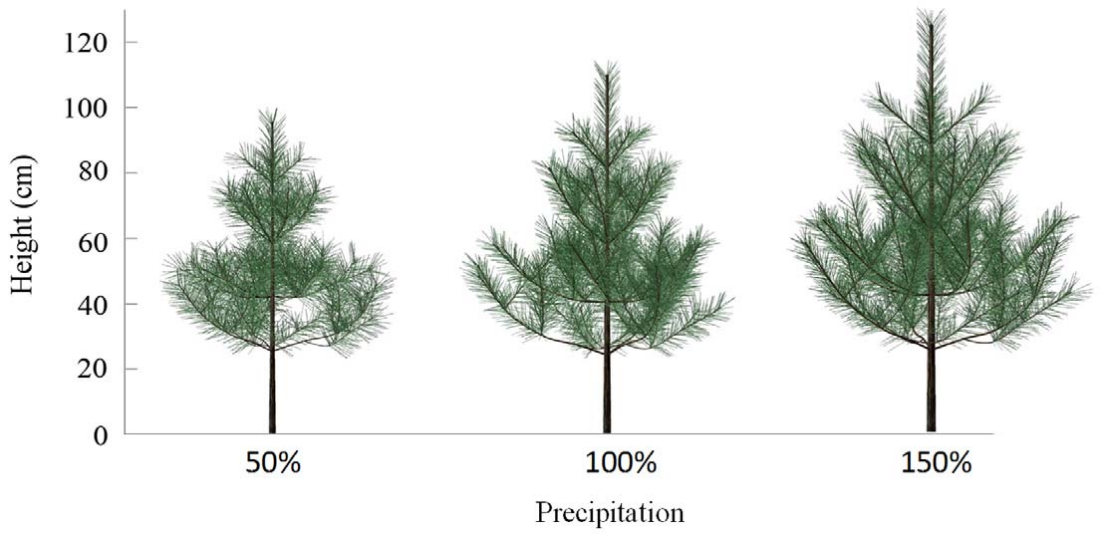
The simulated 3D canopy architecture of six-year-old trees under three precipitation regimes of 50%, 100% and 150% of actual precipitation.

Figure 9 shows the simulated 3D canopy architecture of six-year-old trees under three precipitation regimes of 50%, 100% and 150% of actual precipitation. Their heights are 96 cm, 115 cm, and 126 cm, respectively. Tree height simulated at the actual precipitation was consistent with the observed value of 117.4±10.6 cm.

## Discussion

### Model Performance

In this study, a new module of soil water dynamic was introduced into the GreenLab model. The new adapted GreenLab model is driven by transpiration with water use efficiency for plant biomass affected by temperature, vapour pressure deficit and soil water content. The growth engine of the adapted GreenLab model is water driven through Eq. (1). The model does not simulate the lower hierarchical processes expressing the intermediate steps involved in accumulation of biomass. The underlying processes are synthetically incorporated into one single coefficient defined water-use efficiency (*WUE*). The basis for using Eq. (1) as the core of the model growth engine lies in the conservative behaviour of WUE [37]. The *WUE* value of 4.5 g kg^−1^ for Mongolian Scots pines is obtained by the fitted method in this study. This result is consistent with the range from 3.29 to 6.63 g kg^−1^ calculated for above-ground biomass of *E*. *globulus* in [38]. Almeida *et al*. also gave similar values for fast-growing *E. grandis* plantations in Brazil [39].

### Comparison with Other FSPMs

The aim of most FSPMs is to investigate plant physiological functions over quite short time periods (e.g. several hours, one day or even a few years). These models are not suitable for extending analysis of growth in larger regions or over longer periods because of the large number of parameters to be used and high computational costs. The GreenLab model is adequate for applications at larger scales and also for ecological research by simulating plant biomass production and allocation using empirical equations. Le Chevalier *et al*. proposed a new generic framework for simulating the development of large plant ecosystems using the plant growth engine of the GreenLab model [40]. In our study, we analysed the simulation results related to tree height, basal diameter and biomass of above-ground plant parts for different levels of precipitation.

The high topological complexity and very large number of organs in a tree raise huge difficulties in collecting detailed data at the organ scale. This is why most functional-structural tree models use only aggregated or sampled experimental data as criteria for evaluating their performance in representing the real developmental processes of growth [41]–[43]. Lopez *et al*. also point out that more quantitative validation of models is required to evaluate the accuracy of simulated results [14]. We present here the calibrated and validated processes of an adapted GreenLab model, and the results are shown to be reasonable for Mongolian Scots pine. The model can simulate the variability of plant tree height, basal diameter and biomass fairly well based on automaton and source-sink relationship principles. In recent studies of FSPMs, light is taken as one of the main environmental factors, and 3D models of crown architecture and function are used to simulate light interception [44], [45]. In GreenLab, the level of architectural description needed is that of the topology [24], which needs to be adequately simulated since it has interactions with the functional part of the model (e.g. for ring biomass partitioning, and for computing the demand). As regards the 3D orientations of organs or branches, they have been integrated only for a purpose of visualisation and are not used in the computations. In this simulation study, only organ size in plant architecture changed with different levels of precipitation. Although plant morphological plasticity could be linked to the effects of competition for light, the abundance of the solar radiation in arid and semiarid areas suggests that not light but water was the main limiting environmental factor for plant growth in our study region. Besides, soil water dynamic has seldom been introduced to three-dimensional models for assessing plant growth. L-Peach captured the influence of water stress on growth by introducing a relative water-stress index, which was produced by a sigmoid function [14]. Wu *et al.* proposed a sub-model of plant growth and development with a detailed representation of the root system, in addition to sub-models for C and N cycling in the soil with links to the plant, a soil-water component and a heat-transfer component [46]. The model structure is very complex but above-ground plant growth is simplified. In our study, by incorporating a soil water dynamic module into the GreenLab model, the adapted model allows accurate representation of 3D canopy architecture, morphological and biomass dynamics under the influence of different soil water contents.

### Future Improvements to the Model

Root systems are central to the acquisition of water and nutrients by plants, as root uptake controls actual plant transpiration, water recharge and nutrient leaching to the ground water [47]. GRAAL-CN is one of the first FSPMs to integrate both shoot and root development with similar levels of details [48]. It accounts for plant plasticity in response to resource availability (carbon, nitrogen) but does not take into account water uptake by the roots. However, their model could be a useful source of ideas for future improvements to our model by adding details of root structure and root extension that are lacking in our present version. Similarly, in incorporating processes that take place in the roots, such as water and nutrient uptake into the model, there is an identified need to more faithfully capture the functional dynamics of root-shoot interactions.

The daily water dynamic module in GreenLab is probably the most questionable component, mainly on the grounds that it neglects of effects of stomotal control on plant transpiration. The canopy conductance and boundary layer conductance for Mongolian Scots pines may be added to the original Penman-Montieth equation (see Eq. (2.48) in [49] to calculate tree actual transpiration, instead of Eq. (6). It would be better and have clearer biophysics meaning for soil water dynamic module. Additionally, more validation of the model, especially of the new soil water dynamic module, is needed to evaluate the accuracy of the simulated transpiration.

Another path for improvement is to take into account the effects of source variations on sink satisfaction and the effects of the source-sink ratio on plant architecture through effects on sink numbers and hierarchies [50]. In our experiments, we observed that the number of second- and third-order branches increased with total biomass. Thus, a new version of the model which integrated feedback between sources and sinks could be applied in our future research [16].

In summary, the current version of the model may be improved by incorporating increasing complex descriptions of various facets related to the trees growth and their environmental interactions. However, every increase in model complexity requires more experiments to provide parameters values or other data. Therefore, the prospective value of improvements has to be weighed against the likely availability of the information needed to quantify those improvements, and the gains in accuracy that might be expected from them. The degree of model complexity depends on the purpose for which the model is intended.

### Conclusions

A functional-structural tree model that includes biomass production, partitioning among organs and responses to water availability has been developed for simulating the interaction between plant and environment. This has been calibrated and validated for Mongolian Scots pine based on plant biomass, topological and geometrical descriptions of the plants, soil and meteorological data. The characters of architecture and biomass dynamics of Mongolian Scots pine have then been reproduced and analysed under different levels of water stress using this adapted GreenLab model. The model is expected to perform virtual experiments that predict the behavior of Mongolian Scots pines in other regions with different soils and climates.

## Supporting Information

Appendix S1 **Description of the adapted GreenLab model.**
(DOCX)
