# Supplementary material for: A Functional and Structural Mongolian Scots Pine (Pinus sylvestris var. mongolica) Model Integrating Architecture, Biomass and Effects of Precipitation
Source: PLoS One. 2012 Aug 22;7(8):e43531. doi: 10.1371/journal.pone.0043531 (PMC3425476; doi:10.1371/journal.pone.0043531)
Supplement: Appendix S1 — Description of the adapted GreenLab model. (DOCX) [file pone.0043531.s001.docx]

**Appendix S1: Description of the adapted GreenLab model**

**Ring biomass repartition**

The biomass of internodes comes from their primary growth (corresponding to internode elongation) and from secondary growth (corresponding to the annual growth-ring increment). Primary growth takes place exclusively during the first year while secondary growth does not stop until the branch is dead. The partitioning of ring biomass is described by [1], as shown:

$\left\{\begin{matrix} D_{\text{g}}(i)=\sum_{k=1}^{M} \sum_{m=1}^{i-1} N_{\text{e}}(k,m,i-1)\cdot l(k,m-i+1)\cdot R_{\text{p}}(k) \\ D_{\text{p}}(i)=\sum_{k=1}^{M} \sum_{m=1}^{i-1} N_{\text{a}}(k,m,i-1)\cdot N_{\text{e}}(k,m,i-1)\cdot l(k,m-i+1)\cdot R_{\text{p}}(k) \\ \Delta q_{\text{c}}(k,m,i)=\left(\frac{1-\lambda}{D_{\text{g}}(i)}+\frac{\lambda N_{\text{a}}(k,m-1,i-1)}{D_{\text{p}}(i)}\right)\cdot l(k,m-i+1)\cdot R_{\text{p}}(k)\cdot\Delta Q_{\text{c}}(i) \end{matrix}\right.$ (S1)

where *M* is the maximal physiological age (PA); *N*_e_(*k*, *m*, *i*-1) represents number of growth unit of PA *k*, chronological age (CA) *m* at tree age *i*-1; *l*(*k*, *m-i+*1) represents the length of the growth unit of PA *k* that appears at growth cycle *m*-*i*+1; *R*_p_(*k*) is repartition coefficient for biomass allocation to cambial growth of the growth unit of PA *k* (m^-1^), it is a relative value, with its reference being the growth unit of PA 1; *N_a_*(*k*, *m*, *i*-1) is the number of living needles above a growth unit with of PA *k* and CA *m* at tree age *i*-1; *λ* is a proportion coefficient for blade influence on ring partitioning in [0, 1]; *D*_g_(*i*) and *D*_p_(*i*) represent the plant demand for ring growth at tree age *i* with the uniform and Pressler rules respectively, Δ*q*_c_(*k*, *m*, *i*) is the ring biomass increment of the growth unit of PA *k*, CA *m* at tree age *i*.

**Organ geometry**

The geometry of organs can be obtained according to allometric rules linking biomass and dimension. A needle area is computed from its length *L* and diameter *R* based on the allometric relationship provided for Mongolian Scots pine [2].

*S*=2.57**R*(*L*-0.1167) (S2)

It is assumed that specific leaf weight *ε* is constant during the growth period and can be defined for needles as the ratio between fresh biomass of needles *q*_a_ and leaves surface of needles *S*:

*ε*=*q*_a_(*i*)/*S*(*i*) (S3)

The geometry of the internode is considered as a central cylinder which represents primary growth, with successive ring layers being added during subsequent annual growth cycles. The geometry of the central cylinder is defined by a power function linking cross-sectional area to length, which implies the following relationships between the length *l*_e_(*k*, *i*)、cross-sectional area *s*_e_(*k*, *i*) and biomass *q*_e_(*k*, *i*) of a internode of PA *k* at tree age *i*:

$\left\{\begin{matrix} l_{e}(k,i)=\sqrt{b(k)}\cdot q_{e}(k,i)^{(1+\beta(k))/2} \\ s_{e}(k,i)=\sqrt{1/b(k)}\cdot q_{e}(k,i)^{(1-\beta(k))/2} \end{matrix}\right.$ (S4)

where *b*(*k*) is a scale coefficient and *β*(*k*) is a shape coefficient at PA *k*.

The *x*^th^ layer of the growth unit of PA *k* and CA *m* at tree age *i* is formed at the year *i*-*m*+*x*+1 of tree growth, when the growth unit CA is *m*-*x*-1. So its cross-sectional area is the ratio between the biomass allocated to the ring increment and its length *l*(*k*, *m*-*i*+1):

$s_{\text{c}}(x,k,m,i)=\frac{q_{\text{c}}(k,m-x-1,i-m+x+1)}{l(k,m-i+1)}$, with 1≤*m*≤*i*, 1≤*x*≤*m* (S5)

Thus, the radius of the *x*^th^ growth unit of this axis *r*_c_(*k*, *m*, *i*) is:

$r_{c}(k,m,i)=\sqrt{(s_{e}(k,n-i+1)+\sum_{x=1}^{m}s_{c}(x,k,m,i))/\pi}$ (S6)

**References**

1. Letort V, Cournede P, Mathieu A, de Reffye P, Constant T (2008) Parametric identification of a functional-structural tree growth model and application to beech trees (*Fagus sylvatica*). Functional Plant Biology 35: 951-963.
2. Jiao SR (1982) A method for estimating surface area of Mongolian Scots pine plantations needles. Liaoning Forest Science and Technology 189: 21-23. (in Chinese)
